# Integration of Hot Tube Gas Forming and Die Quenching of Ultra-High Strength Steel Hollow Parts Using Low Pressure Sealed-Air

**DOI:** 10.3390/ma15041322

**Published:** 2022-02-10

**Authors:** Ali Talebi-Anaraki, Tomoyoshi Maeno, Yuta Matsubara, Ryohei Ikeda, Ken-ichiro Mori

**Affiliations:** 1 Department of Mechanical Engineering, Materials Science, and Ocean Engineering, Graduate School of Engineering Science, Yokohama National University, Yokohama 240-8501, Kanagawa, Japan; matsubara-yuta-fk@ynu.jp (Y.M.); ikeda-ryohei-vb@ynu.jp (R.I.); 2 Division of Systems Research, Faculty of Engineering, Yokohama National University, Yokohama 240-8501, Kanagawa, Japan; maeno-tomoyoshi-yf@ynu.ac.jp; 3 Department of Mechanical Engineering, Toyohashi University of Technology, Toyohashi 441-8580, Aichi, Japan; mori@plast.me.tut.ac.jp

**Keywords:** tube forming, hot forming, gas forming, bulging, low pressure, die quenching

## Abstract

A low pressure sealed-air hot tube gas forming process of ultra-high strength steel tubes was developed not only to change the cross-section of the hollow products by bulging but also to increase the strength of components. Gas-formed components are typically formed by a controlled-gas pressure with extremely high internal pressure, which leads to affected production costs and safety. Moreover, compressing the gas with high pressure requires high energy during its preparation. Therefore, to simplify the internal pressure controlling system and improve the safety factor in gas forming processes, the sealed-air tubes are formed with a quite low initial pressure. The pressure of the sealed air increased with increasing temperature of the air inside the resistance-heated tube, and the bulging deformation was controlled only by axial feeding. The effects of the initial pressure and heating temperature on the bulging deformation and quenchability of the tubes, and the effect of the starting time of axial feeding on the bulging behavior were examined. Consequently, ultra-high strength steel bulged parts were produced even in low initial internal pressure and with the rapid heating of the tubes.

## 1. Introduction

The requirements to reduce the overall vehicle weight and consequently fuel consumption to meet environmental regulations whilst satisfying consumer demand for part quality and collision safety have presented the automotive industry with various challenges. Therefore, the development and application of lightweighting materials, such as high strength steels, have increased mainly for body-in-white parts. Although utilizing hollow parts can reduce the weight of products, joining processes of the separated parts are increasingly required. Resistance spot welding is commonly used to join steel sheets for automobile body panels; however, the joining process can decrease the final quality and stiffness of the products. Therefore, utilizing tubes such as hollow parts has become attractive.

Tube hydroforming is a forming process to form complex-shaped hollow products with closed cross-section geometry from tubes by using internal pressure and axial feeding [1]. To avoid fracture and thinning during the hydroforming of tubes, the material is axially fed along the tube length; however, by increasing the axial force over a critical limit, which is not compatible with the internal pressure, the tube tends to wrinkle [2]. Therefore, designing a suitable pressure and axial feeding path is critical for successfully hydroformed the tubes. To form the small corner radius the required fluid pressure is high and leads to bursting and the reduction in the material feeding. Nikhare et al. [3] simulated the high and low pressure hydroforming of a TRIP 780 MPa high strength steel tube to determine the effectiveness of lower internal pressure in tube hydroforming. Nikhare et al. [4] prevented the bursting of stainless steel tubes in low pressure hydroforming by optimizing the internal pressure and die closing force. The results confirmed that the required internal pressure and die closing force for low pressure tube hydroforming is much less than that of high pressure tube hydroforming. Chu et al. [5] predicted the effect of internal pressure on the springback during low pressure tube hydroforming. However, the hydroforming process leads to rupture or folding defects for low-ductility materials such as high strength steel tubes.

To improve the formability of low ductility materials such as aluminum and magnesium alloys at room temperature, warm hydroforming has been developed. Liu et al. [6] investigated the effects of axial feeding and temperature on warm hydroforming of an AZ31B magnesium alloy tube. Their results demonstrated that the wrinkles with a certain shape and amount can be useful to increase the expansion ratio in warm hydroforming. Kim et al. [7] analyzed the heat transfer between the tube and the die in warm hydroforming of an AA6061 aluminum extruded tube and found that the bulging height increases at higher forming temperatures. Yi et al. [8] proposed a combined heating system using an induction coil and a heating element for warm hydroforming of AA6061 aluminum alloy tubes. However, the heating temperature is generally limited below 300 °C to prevent evaporation of the fluid media such as oil and water, and such low temperatures are not enough for high strength steel tubes. Therefore, the fluid medium in hydroforming was replaced with gas to eliminate the limitation of the heating temperature in tube forming.

He et al. [9] studied the free bulging test at elevated temperatures to evaluate the formability in gas forming of AA6061 aluminum alloy tubes. The results indicate that the maximum expansion ratio reached the maximum value of about 86% at 425 °C. Maeno et al. [10] investigated the effects of different current densities on the bulging behavior of AA6063 aluminum alloy tubes using internal air pressure and resistance heating. To prevent the temperature drop around the tube ends, stainless steel rings were inserted between the tube and electrodes. Maeno et al. [11] optimized the axial feeding curves during the hot gas bulging process of aluminum alloy tubes. Their results revealed that the quality of bulged tubes depended on the feeding parameters. Trân et al. [12] developed a superplastic hot gas forming process for titanium tubes to manufacture helically shaped flex hollow parts without forming defects by defining an optimized forming pressure-time profile. Talebi-Anaraki et al. [13] utilized flame heating for dieless gas forming of aluminum alloy tubes to investigate the bulging behavior in the local heating area. Talebi-Anaraki et al. [14] improved the formability of tubes in gas forming by utilizing pulsating pressure paths during oscillating heating of aluminum alloy tubes. Their results indicate that pulsating the gas pressure during forming led to the production of more complex components with uniform thickness distribution. Tang et al. [15] proposed an electropulsing assisted pulsating gas forming to form CP-Ti tubes using argon pressure. It was found that the relative load and internal pressure fluctuate periodically along with an oscillation of temperature. The gas forming processes have high flexibility; however, for gas pressure, it is not easy to control the deformation behavior. 

In hot stamping, quenchable steel sheets are heated up to approximately 900 °C for the transformation into austenite phase. Subsequently, the heated sheets are rapidly cooled just after the end of forming by holding with dies at the bottom dead center of a press in a process called die quenching to produce ultra-high strength components gained by the martensite transformation during die quenching [16]. Gas forming is also utilized for quenchable steel tubes not only to produce the ultra-high strength steel hollow components, but also to omit the additional heat treatments. Vadillo et al. [17] investigated the gas forming of high strength steel and stainless steel tubes using resistance heating between 500 and 900 °C. Paul and Strano [18] examined the influences of forming conditions on the press hardenability and forming shape in the gas forming of steel tubes. Their results revealed that the hardness of the formed tube is locally dependent on the pressure–temperature curve, tube pre-heating temperature, and the tool temperature. Neugebauer and Schieck [19] developed a gas forming process with a maximum pressure of 80 MPa and a maximum temperature of 1000 °C for tube press hardening with integrated heat treatments to form high-strength products. Bach et al. [20] heightened a pre-bent steel tube with the combination of gas forming and conductive heating. Winter et al. [21] investigated the influence of local cooling rates on the mechanical properties of the quenching and partitioning-steels in gas forming with the forming pressure of 70 MPa. However, the required internal pressure in the gas forming of steel tubes is still high. The required pressure in gas forming processes is comparatively lower than that of hydroforming, while it leads to an explosive-bursting noise, even in working pressures less than 70 MPa. Moreover, the high pressure equipment is costly and requires pressure control systems. Consequently, utilization of low pressure in tube gas forming processes can be attractive. 

Maeno et al. [22] fabricated a V-shaped ultra-high strength steel hollow part by compression of a sealed tube with resistance heating. In this stage, an internal low pressure as a reaction force is applied at the beginning of the stamping process; however, the pressure is automatically increased to 4–5 MPa by a reduction in the internal volume of the tube, and not by bulging. 

The effect of the weld zone on deformation behavior in the compression forming of steel tubes such as V-shaped compression is too low due to local deformation in the contact area. In the case of bulging of steel tubes, the generated hoop stress by internal pressure is uniform; however, the mechanical properties of the steel tube is not uniform due to the inevitable existence of a heat affected zone and thus the bulging deformation of the welded steel tubes is found to be different from extruded aluminum alloy tubes. Omar et al. [23] investigated the effect of bulge ratio on the deformation behavior and fracture location during hydroforming of electric resistance welded drawing quality steel tubes. Their results indicate that the thickness imperfection and material inhomogeneity affect the location of a crack during welded tube hydroforming. Omar et al. [24] studied the forming limit diagrams during the hydroforming of welded steel tubes. The simulations results confirmed that the presence of a weld zone reduced the hydroformability of the steel tubes. Moreover, the low emissivity of aluminum alloys results in a low cooling rate in the atmosphere compared with the steel tube. However, the feasibility of die quenching of bulged ultra-high strength steel tubular components using low internal pressure is not yet clear. Therefore, it is desirable to not only improve the bulging deformation, but also to increase the cooling rate during the bulging of steel tubes for bulging with low pressure sealed-air pressure.

In the present study, a low pressure sealed-air hot gas forming process of tubes was developed to produce ultra-high strength steel hollow components. The internal pressure control was simplified by sealing the air in the tube during forming with axial feeding, and the bulging deformation and die quenchability were investigated. 

## 2. Hot Tube Gas Forming and Die Quenching of Ultra-High Strength Steel Hollow Parts Using Sealed Air and Resistance Heating

### 2.1. Utilization of Low Pressure Sealed Air in Hot Tube Gas Forming

**Figure 1:**
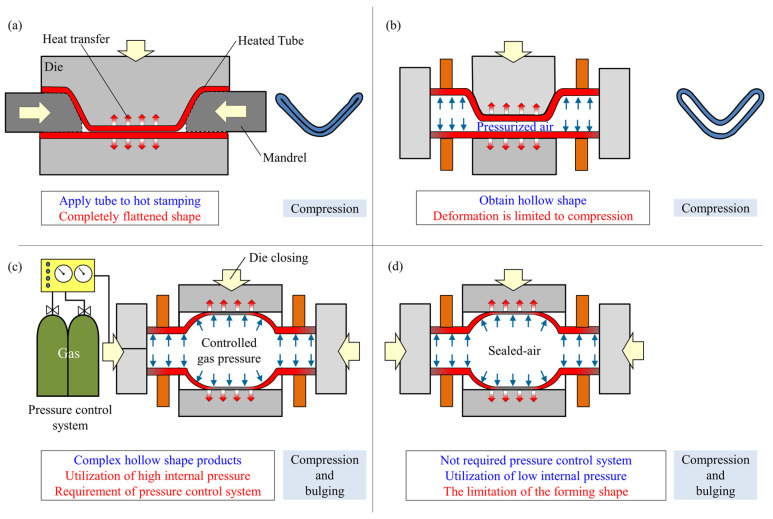
Hot forming of tubes with die quenching: (**a**) hot stamping of tubes without internal pressure; (**b**) hot stamping of tubes with internal pressure; (**c**) bulging of tubes using controlled gas pressure; and (**d**) bulging of tubes using low pressure sealed-air tube.

As it is shown in Figure 1a, since the contact pressure at the interface between the tube and die is low without internal pressure, compression up to complete flattening is required to obtain sufficient die quenchability. However, by applying the internal air pressure in the tube, hollow shape products can be obtained by the increase in contact pressure as shown in Figure 1b. However, the importance of bulging and volume increase in the tubes is remarkable for forming complex-shaped hollow products, especially those with curvature or small round corners in a cross-section. In gas forming processes, the tubes are directly pressurized by an air source; however, it is difficult to simultaneously control the heating temperature and internal pressure during a short time of bulging, and thus an accurate pressure control system is required as shown in Figure 1c. Moreover, it is not easy to change the amount of the gas media rapidly compared with non-compressive fluid media. Consequently, as it is shown in Figure 1d, the control scheme in gas forming is simplified by using the air-filled sealed tube due to the adjustment of the range of pressure by the initial air pressure in the sealed tube before forming. However, it is difficult to directly control the start of bulging using sealed-air, and to control the bulging behavior the timing of axial feeding becomes important. Therefore, the low pressure sealed-air gas forming of a quenchable steel tube using axial feeding was developed not only to simplify the controlling system, but also to improve the bulging deformation and die quenchability. 

**Figure 2:**
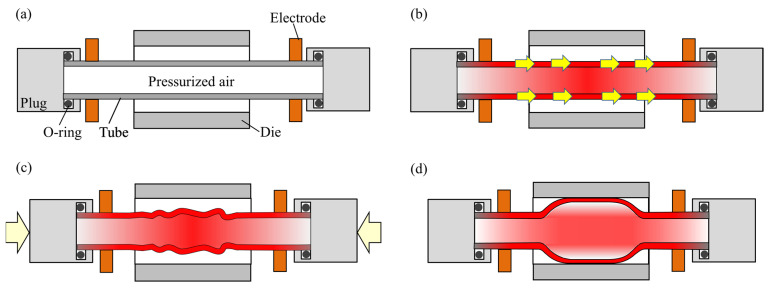
Sequence of sealed-air hot tube gas forming using resistance heating: (**a**) setup and charge with compressed air; (**b**) resistance heating (pressure increasing); (**c**) start of axial feeding; and (**d**) bulging and die quenching.

The sealed-air hot tube gas forming process of ultra-high strength steel hollow parts using axial feeding and resistance heating is shown in Figure 2. First, the sealed quenchable steel tube is set to both electrodes, and is charged with pressurized air. Then, the tube is resistance-heated to decrease the flow stress and to austenitize. To prevent thinning, the tube is compressed in the axial direction during heating. Finally, the tube is bulged and die-quenched.

**Figure 3:**
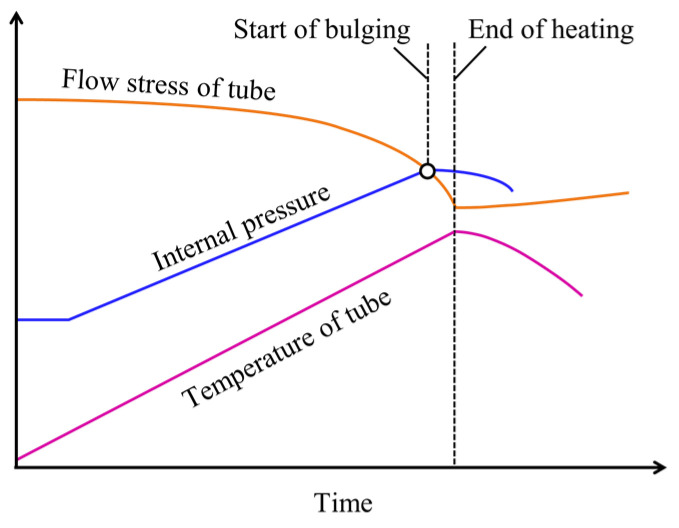
Variations in process parameters during sealed-air hot tube gas forming using resistance heating.

The variations in the process parameters during sealed-air hot tube gas forming using resistance heating are shown in Figure 3. The temperature of the tube immediately increases from the start of resistance heating, and the flow stress of the tube decreases, whereas the rise of internal pressure is delayed from that in tube temperature. The tube is bulged by the decreases in flow stress and increases in internal pressure.

The internal pressure *P_h_* just before bulging of the tube follows the ideal gas rule and is represented as:

(1) $$P_{h}=\left(P_{0}+0.1\right)\frac{T_{1}+273}{T_{0}+273}-0.1$$

where *P*_0_ is the initial internal air pressure, *T*_0_ and *T*_1_ are the temperatures in degree Celsius of the internal air of the tube before and after heating, respectively.

### 2.2. Principles of Sealed-Air Hot Tube Gas Forming Using Axial Feeding and Resistance Heating

**Figure 4:**
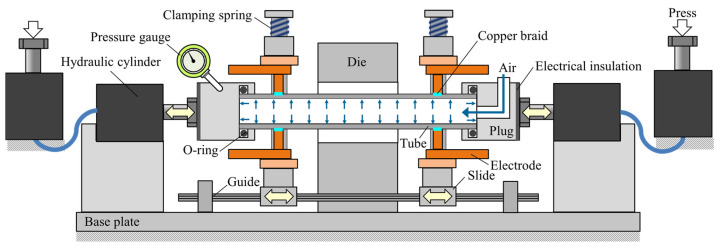
Experimental apparatus for sealed-air hot tube gas forming using resistance heating and axial feeding by press slide.

**Figure 5:**
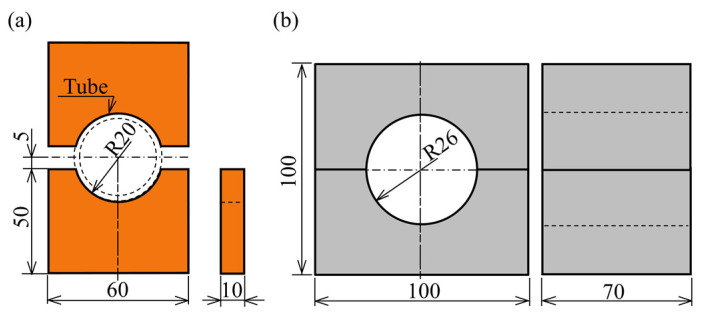
Dimensions of tools for sealed-air hot tube gas forming using axial feeding and resistance heating: (**a**) electrodes; (**b**) dies (in mm).

The experimental apparatus for sealed-air hot tube gas forming was designed as shown in Figure 4. The apparatus was composed of resistance heating electrodes, sealing, pressurizing, and axial feeding units to bulge the middle of a cylindrical quenchable steel tube. The setup was installed in an 800 kN CNC servo press (SDE-8018, Amada Co., Ltd., Isehara, Japan) to drive the axial feeding unit by a press slide. Both ends of the sealed tube having 200 mm length in the longitudinal direction were sandwiched between the copper electrodes under an average pressure of 2 MPa by clamping springs. The dimensions of the copper electrodes and forming die are shown in Figure 5. To heat the tube uniformly and obtain the uniform distribution in electrical contact resistance, tin coated flat copper braids were inserted at the interface between the electrodes and tube to increase the fitting between them. The contact length of the electrodes was 80% of the circumference of the tube. To seal the tube and prevent leakage, rubber O-rings were inserted into the grooves inside both plugs. An open/close valve was installed in one side of the sealing plug to trap the initial internal pressure inside the sealed tube before the start of heating, and the pressure during the forming process was measured by a Bourdon type pressure gauge to omit the noise by resistance heating. To reduce the thickness reduction in the bulged tube, both ends of the tube were compressed by axial feeding during heating. The servo press slide pushed the vertical hydraulic cylinder to move the horizontal one to simplify the equipment for axial feeding the tube compared with the electric motor drive.

The forming die was made of carbon steel of S50C in JIS. The clamped tube is not in contact with the forming die during resistance heating. To prevent bursting the tube in the forming die, the inner diameter of the die was set to 52 mm, as the tube burst at attaining 55 mm in outer diameter in free bulging without axial feeding. The conditions utilized for sealed-air hot tube gas forming are shown in Table 1. To verify the repeatability of the experiments, the experiments for each of the conditions were performed three times.

**Figure 6:**
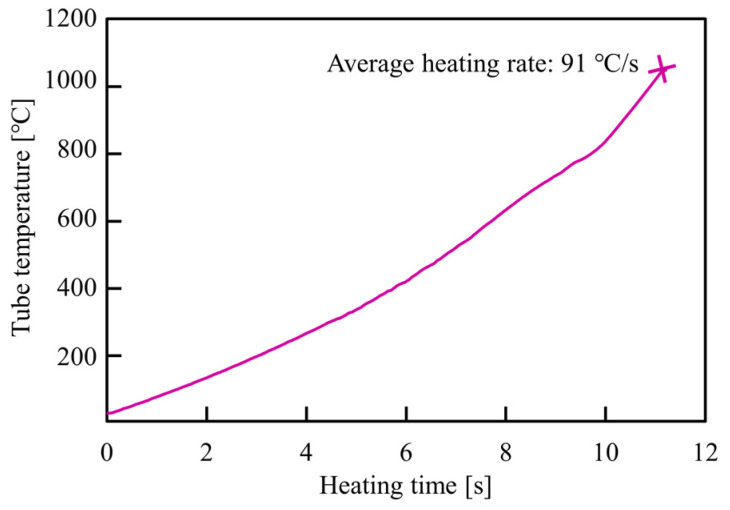
Temperature history of center of the tube during resistance heating.

The quenchable steel welded tube with a carbon content of 0.19 wt% and a small amount of boron was employed for the experiments, which is almost similar to the 19MnB5 grade steel. The chemical composition of the as-received tube is provided by the manufacturer (Nippon steel, Tokyo, Japan) and is given in Table 2. The austenite transformation temperature (Ac3) of the as-received steel tube is about 820 °C, and the tube was heated up to almost 1050 °C due to the rapid heating time by resistance heating. The temperature history of the center of the tube during resistance heating is shown in Figure 6. The rise in heating temperature is effective to increase the internal pressure and to decrease the flow stress of the tube. The outer diameter and thickness of the tube were 38.1 mm and 1.6 mm, respectively. The weld bead of the as-received tube was already removed, and the Vickers hardness of the as-received tube was 208 HV10. 

**Figure 7:**
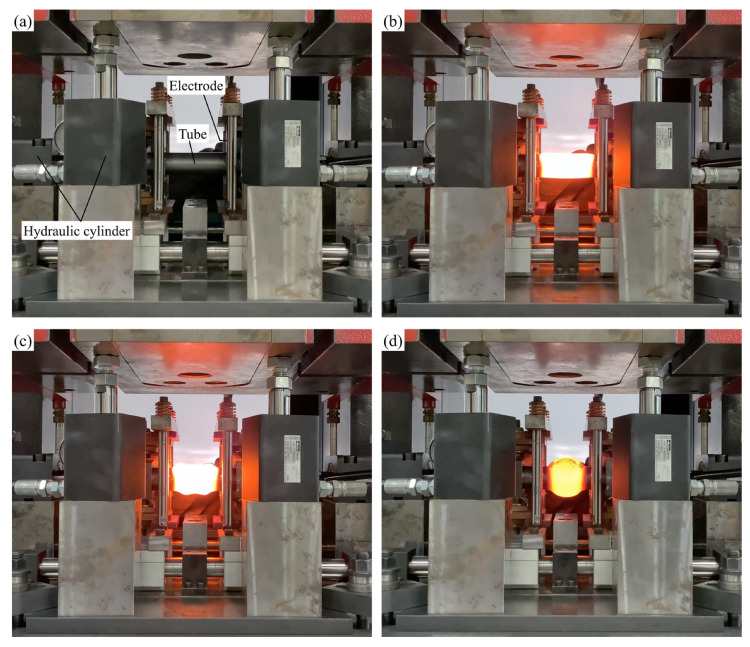
Tube deformation scenes of sealed-air hot gas forming without forming dies: (**a**) setup; (**b**) during resistance heating; (**c**) just after axial feeding; and (**d**) end of bulging.

The tube deformation scenes of sealed-air hot gas forming without forming dies are shown in Figure 7. The sealed-air tube is clamped between the electrodes and resistance-heated. Then, the press slide is pushed downward by the hydraulic cylinders, and axial feeding is applied from both ends of the tube. Finally, the tube is freely bulged by the sealed-air internal pressure. The temperature was measured from the side by a two-color radiation thermography with an accuracy of ±1% (Thermera-NIR2, Nobby Tech. Ltd., Tokyo, Japan), and the effect of the change in surface emissivity by oxidation was eliminated [25].

## 3. Deformation Behavior of Low Pressure Sealed-Air Hot Tube Gas Forming Using Axial Feeding

### 3.1. Influences of Initial Pressure and Heating Temperature on Bulging Deformation

**Figure 8:**
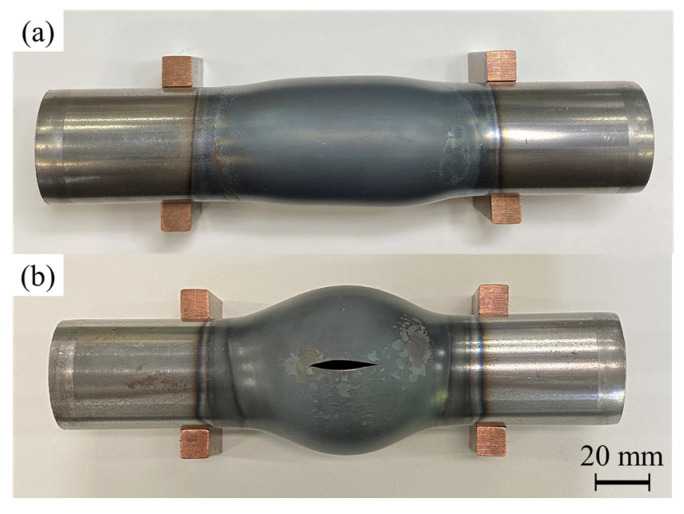
Bulged tubes with: (**a**) *p*_0_ = 1.9 MPa and; (**b**) *p*_0_ = 2.5 MPa and heating time of 11.5 s without axial feeding from top-view.

The bulged tubes with *p*_0_ = 1.9 and 2.5 MPa, a current density of 30 A/mm^2^, and a heating time of 11.5 s are illustrated in Figure 8, where *p*_0_ is the initial internal air pressure. For *p*_0_ = 1.9 MPa, the tube was slightly bulged and it was not enough to burst the tube, i.e., the deformation was stopped due to insufficient internal pressure. However, the bulging ratio becomes remarkable when *p*_0_ = 2.5 MPa. The tube length even without axial feeding becomes shorter by bulging deformation.

**Figure 9:**
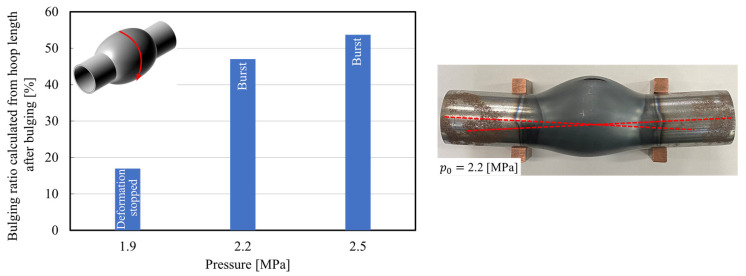
Bulging ratio of center of tubes for *p*_0_ = 1.9, 2.2, and 2.5 MPa.

The effect of the initial pressure on the bulging ratio is shown in Figure 9. The bulging diameter was obtained from the hoop length at the bursting portion due to non-axisymmetric bulging. In the case of the low initial pressure, the tube is bent due to the difference in the temperature distribution of the tube during bulging and the bulging diameter is decreased compared with high initial pressure.

**Figure 10:**
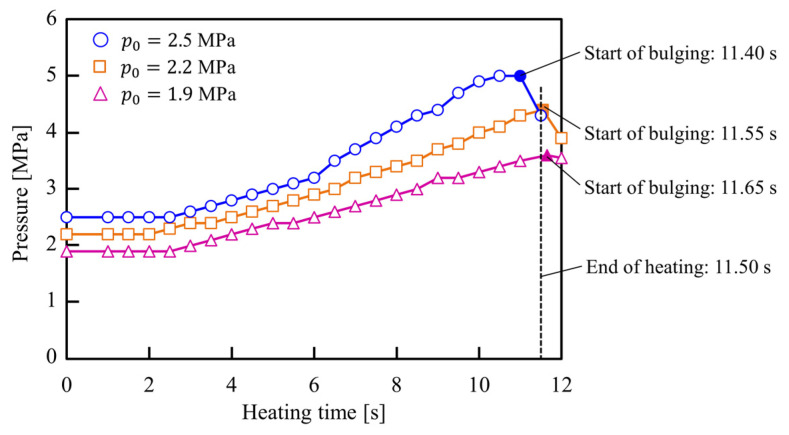
Internal pressure histories of sealed-air during resistance heating for *p*_0_ = 1.9, 2.2, and 2.5 MPa.

**Figure 11:**
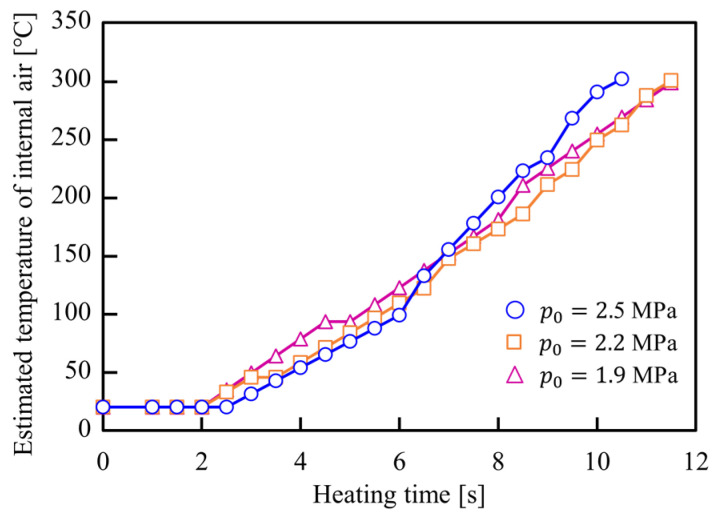
Estimated internal air temperature histories of sealed-air during resistance heating for *p*_0_ = 1.9, 2.2, and 2.5 MPa.

The internal pressure and estimated internal air temperature histories of the sealed air during resistance heating are illustrated in Figure 10 and Figure 11, respectively. The internal air pressure in the sealed tube automatically increases with the increase in the temperature of the air inside the heated tube. Therefore, the sealed-air pressures just after the end of heating were increased from *p*_0_ = 1.9 and 2.5 MPa to *p* = 3 and 5 MPa, respectively. Moreover, the temperature of the sealed-air just after the end of heating was calculated from Equation (1) to be approximately 300 °C, which indicates an almost equal internal air temperature for the various initial internal pressures.

**Figure 12:**
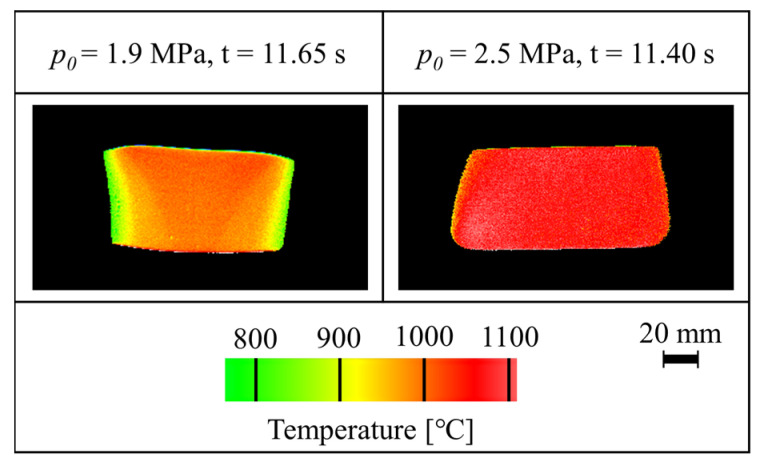
Temperature distributions of the tube just before bulging for *p*_0_ = 1.9 and 2.5 MPa.

The temperature distributions of the steel tubes just before bulging are shown in Figure 12. According to *p*_0_ = 2.5 MPa, the bulging time was 11.40 s from the start of heating, which was approximately the same as the end of heating. In this case, the bulging deformation was finished before the end of heating and a uniform temperature distribution was obtained. The bulge height of the side surface of the tube was approximately equal to its opposite surface, which indicated the uniformity of the temperature along the tube circumference. On the other hand, in the case of low pressure of *p*_0_ = 1.9 MPa, the bulging deformation was started at 11.65 s from the start of heating. Therefore, the tube bulged after the end of heating and the obtained temperature distribution was non-uniform due to the immediate temperature drop of the tube by thermal convection.

**Figure 13:**
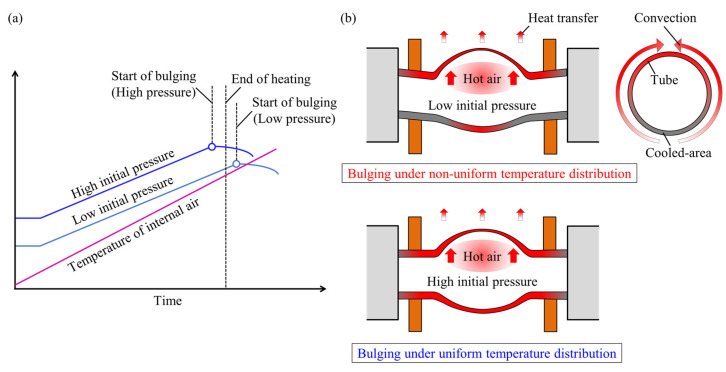
(**a**) Variations in process parameters with low and high initial pressures and (**b**) the difference of the mechanism of deformation behavior of tubes with low and high initial pressures.

The difference in the mechanism of deformation behavior of tubes with low and high initial pressures are shown in Figure 13. Although the initial sealed-air pressure automatically increases with an increase in temperature, in the case of utilization of low initial pressure the bulging of tubes starts after the end of heating, whereas by increasing the initial pressure the bulging begins before the end of heating with an increase in the internal pressure to a pressure of about 5 MPa. Subsequently, the internal pressure slightly decreased due to the increase in internal volume of the tube after the start of the bulging. Moreover, in the case of utilizing high initial pressure, the bending of the tube was prevented due to the uniform temperature distribution of the tube during bulging.

**Figure 14:**
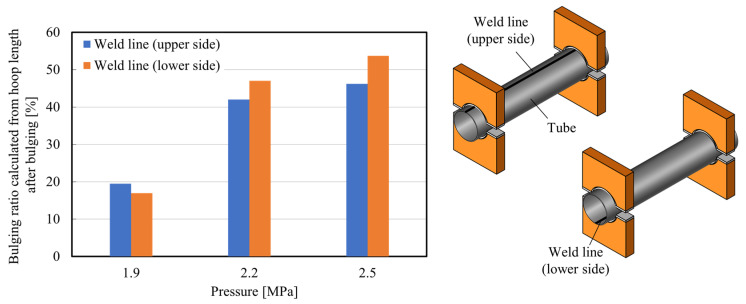
The bulging ratio of the center of the tubes with different weld line positions for *p*_0_ = 1.9, 2.2, and 2.5 MPa and heating time of 11.5 s.

**Figure 15:**
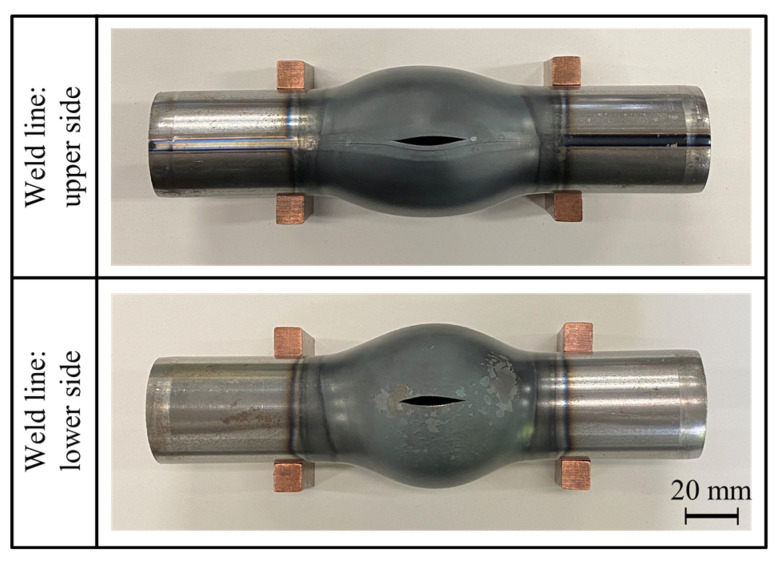
Bulged tubes with different weld line positions for *p*_0_ = 2.5 MPa from top-view.

Figure 14 shows the effect of the weld line position on the bulging ratio of the bulged tubes for *p*_0_ = 1.9, 2.2, and 2.5 MPa. By locating the weld line at the lower side, the tube expansion rate was slightly increased. Although the effect of the weld line on the bulging behavior is slight, the rupture position occurs at the upper side of the steel tube regardless of the position of the weld line due to the convection of the hot air pressure to the upper side as shown in Figure 15; whereas the rupture position in hydroforming tends to occur in the vicinity of the weld of the tube [26].

### 3.2. Influence of Axial Feeding on Bulging Deformation

**Figure 16:**
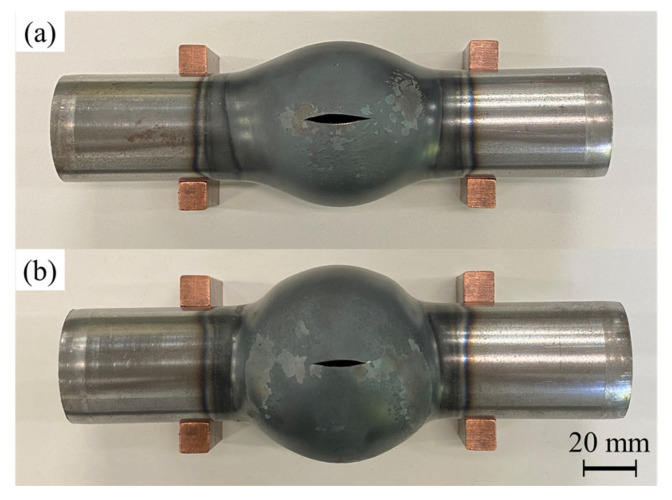
Bulged tubes (**a**) without and (**b**) with 15 mm of axial feeding for *p*_0_ = 2.5 MPa and heating time of 11.5 s from top-view.

Figure 16 shows the bulged tubes with and without axial feeding for *p*_0_ = 2.5 MPa and heating time of 11.5 s. The forming process led to a significant increase in tube expansion rate by axially compressing the tube.

**Figure 17:**
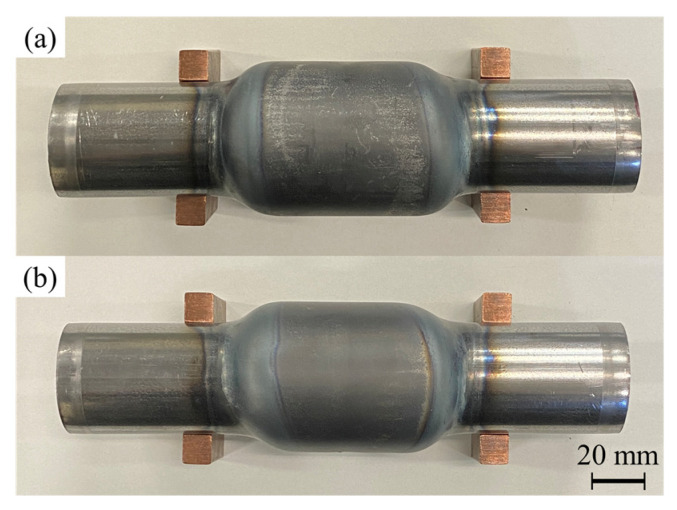
Formed tubes (**a**) without and (**b**) with 15 mm of axial feeding for *p*_0_ = 2.5 and heating time of 11.5 s in forming dies from top-view.

To investigate the effect of the axial feeding, the bulge diameter was fixed by forming dies to compare the thickness distribution of the tube with the same bulging ratio. The formed tubes with and without axial feeding for *p*_0_ = 2.5 MPa are shown in Figure 17. The velocity of axial feeding was 25 mm/s; therefore, the axial feeding is carried out in 0.6 s for *s* = 15 mm. Since the internal diameter of the forming dies is lower than the results of the free bulging, the bursting is prevented in both conditions. In the case of utilizing forming dies, the bulging length of the tubes is increased compared with free bulging results due to the continuation of the bulging deformation after contact of the center of the tube with the dies. Although the obtained bulging diameters of the formed tubes are the same as the diameter of the forming dies, the bulging lengths of the tubes are slightly different. The bulging length is defined as the length of the bulged area above 2% expansion of the initial diameter of the tube, which is equal to 76 mm and 79 mm for the formed with and without axial feeding, respectively. 

**Figure 18:**
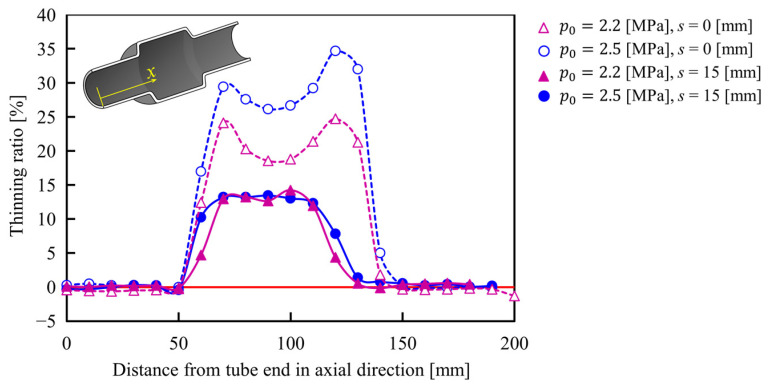
The thinning ratio of the tube, with and without axial feeding, for *p*_0_ = 2.2 and 2.5 MPa and heating time of 11.5 s in longitudinal direction.

The bulged tubes, with and without axial feeding, were cut by a diamond bandsaw and the thickness distribution was measured by a micrometer caliper. The thinning ratio in the longitudinal direction of the tube, with and without axial feeding, is shown in Figure 18. The results showed that the axial feeding prevented the thinning of the tube in the deformation area by providing better material flow during the forming process.

**Figure 19:**
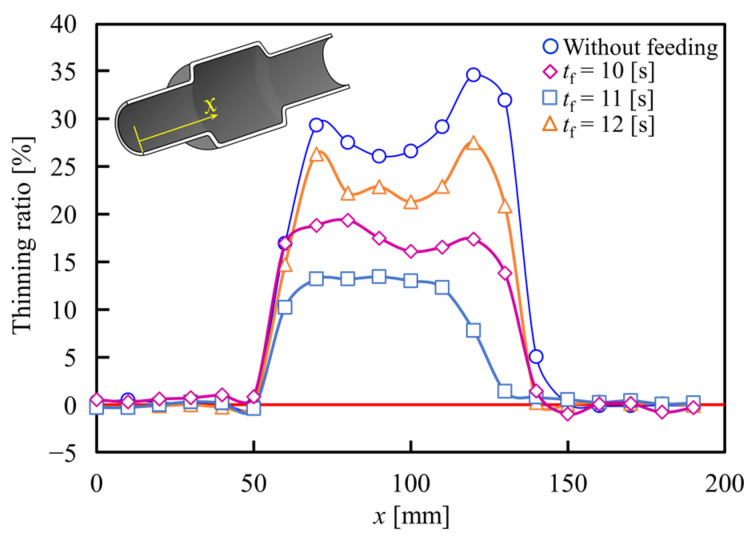
Relationship between starting time of axial feeding and thinning ratio of formed tubes for *p*_0_ = 2.5 MPa and *s* = 15 mm.

**Figure 20:**
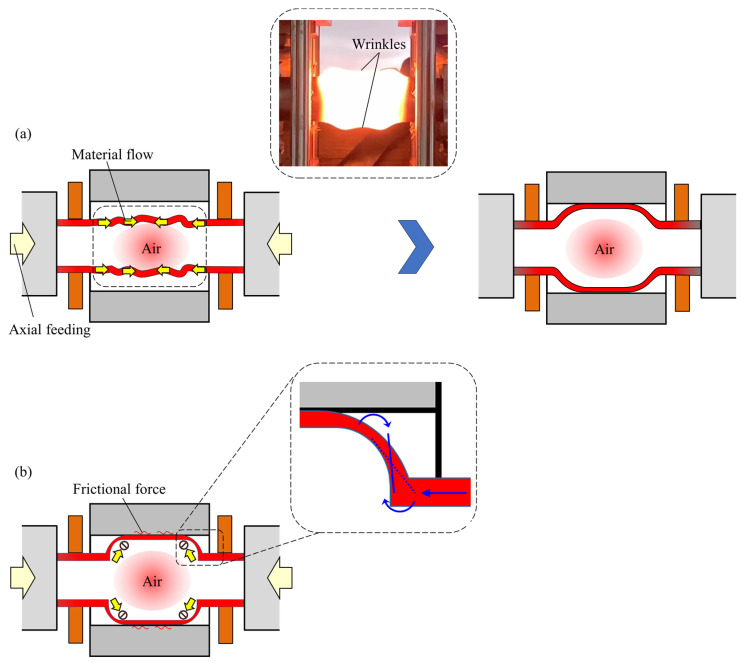
Schematic of prevention of thinning by axial feeding (**a**) before and (**b**) after bulging.

Although the axial feeding of the steel tubes prevents thinning by material flow, it is held by the frictional force generated during contact between the tube and tools surfaces. Therefore, the appropriate time of the axial feeding becomes an important factor that influences the bulging deformation of the tube. The relationship between the starting time of axial feeding and the thinning ratio of the formed tubes for *p*_0_ = 2.5 MPa and axial feeding of 15 mm is shown in Figure 19. By applying early axial feeding at 10 s after the start of heating, the bulging is started earlier than without axial feeding and the material flow led to wrinkling of the tube; however, the fed material is stocked at the ends of the tube and cannot completely flow to the bulging deformation area, and thus the effect of axial feeding is reduced. In the case of applying axial feeding 11 s after the start of heating, the thinning of the tube was significantly suppressed when compared with no axial feeding due to the control of the bulging behavior caused by axial feeding before the start of tube expansion. However, by increasing the start time of feeding to 12 s, the suppression of the wall thinning became small due to the prevention of the material flow by frictional force as shown in Figure 20. The angle of the transition zone of the *t_f_* =10 and 12 are 21° and 33°, respectively. The late feeding is led to the lifting of the transition zone, and it is found that the late axial feeding induces the folding at the transition zone.

## 4. Improvement of Die Quenchability of Tubes in Low Pressure Sealed-Air Hot Tube Gas Forming

### 4.1. Influence of Sealed-Air Hot Tube Gas Forming on Hardness Distribution

**Figure 21:**
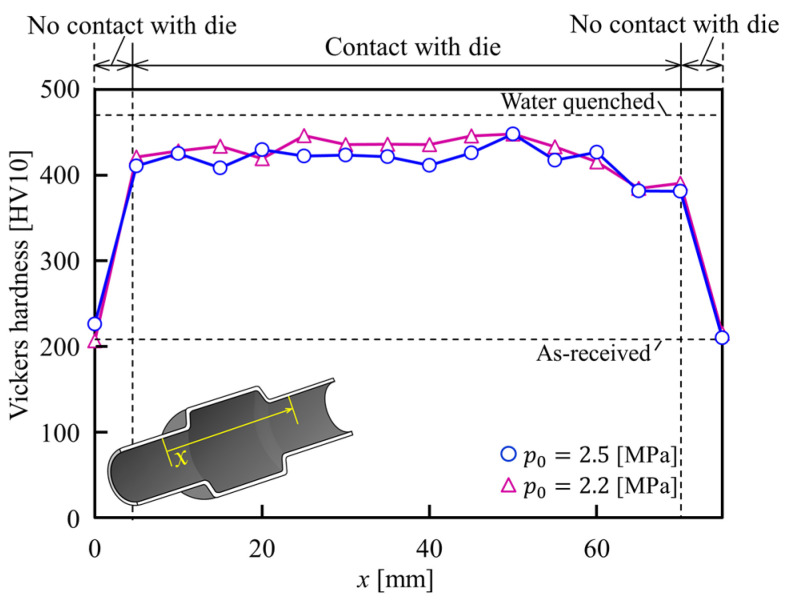
Vickers hardness distributions of formed tubes for *p*_0_ = 2.2 and 2.5 MPa in axial direction.

The Vickers hardness distributions of the axial direction of the gas formed tubes for *p*_0_ = 2.2 and 2.5 MPa are shown in Figure 21. The Vickers hardness of the bulged tube was nearly about 460 HV10, and reached that of a water-quenched tube due to the rise in heat transfer and cooling rate at the interface between the tube and die. Therefore, it can be inferred that the hardening of the formed tubes is gained by sealed-air hot tube gas forming even in low internal pressure.

**Figure 22:**
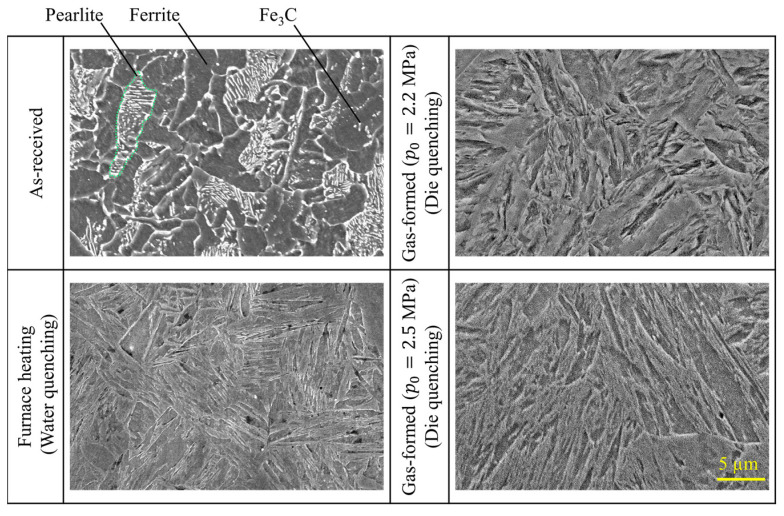
Microstructures at center of formed tubes.

The microstructures at the center of the formed tubes were analyzed by scanning electron microscopy (SEM; JSM-5510, JEOL Ltd., Tokyo, Japan) as shown in Figure 22. At first, the mounted samples were polished to a reflective surface with emery papers and were subsequently lapped with a 1 µm and 0.3 µm alumina powder. To observe the microstructures, the polished surfaces were etched with a 3% Nital solution. The microstructure of the as-received tube consists of the striped area of pearlite, dark area of ferrite, and small precipitates of cementite. The results revealed that the steel tube was heated above the austenitizing temperature, and then was rapidly cooled by contacting with dies during bulging to ensure a martensitic transformation, even for the comparatively short heating time of resistance heating [27].

### 4.2. Measuring Technique of Tube Temperature during Die Quenching Inside Forming Dies

**Figure 23:**
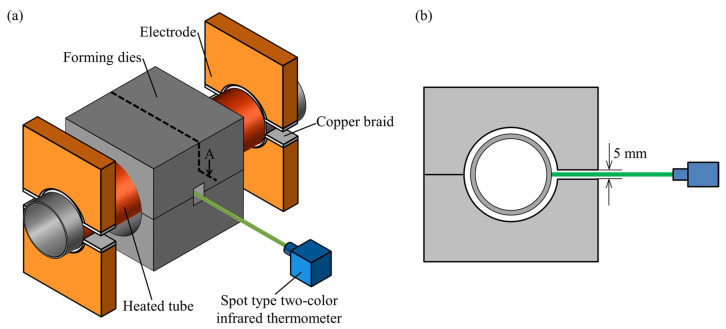
Measurement of the temperature of the tube inside the forming dies using a two-color infrared thermometer: (**a**) schematic and; (**b**) cross-section of tools.

**Figure 24:**
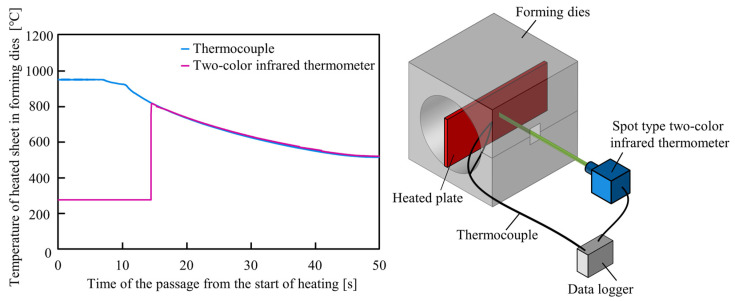
Verification of accuracy of temperature measuring technique without internal pressure.

In order to measure the temperature of the tube inside the forming dies, a small hole with a diameter of 5 mm is drilled in the die and the temperature is measured with a spot type two-color infrared thermometer (CT series, Optris GmbH, Berlin, Germany) as shown in Figure 23. It is not easy to use infrared thermometers in resistance heating due to the changes of the emissivity by the growth of oxidation layers. Moreover, measuring the temperature through the deep hole intensifies the surface emissivity changes. Therefore, the two-color infrared thermometer without emissivity calibration was suitable for measuring the temperature in sealed-air hot tube gas forming even through the small holes. Moreover, the utilized two-color infrared thermometer can measure the temperature above the 5% spot area. To check the accuracy of the measuring technique, the temperature of a heated steel sheet was measured with the two-color infrared thermometer through the die hole and the result was compared with the result obtained by a thermocouple as shown in Figure 24. There is a good agreement between the two measured temperatures.

### 4.3. Cooling Behavior in Forming Dies

**Figure 25:**
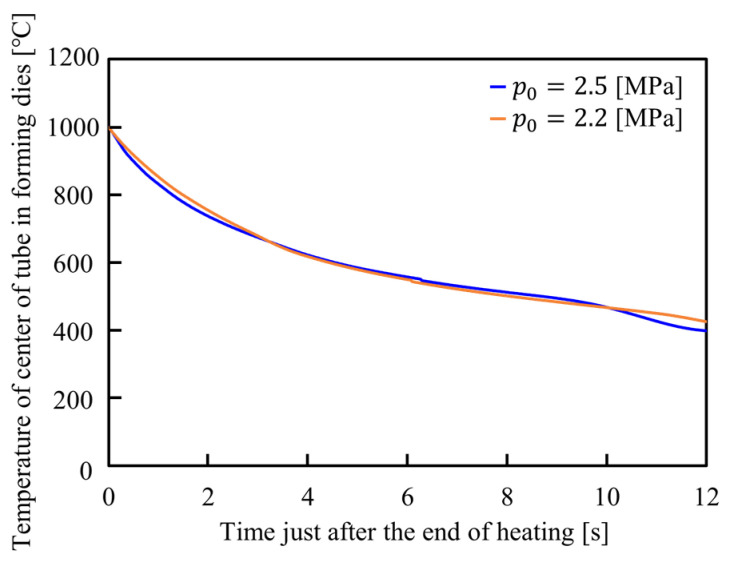
Temperature distributions of the center of the formed tubes just after the end of heating inside forming dies.

The temperature distributions of the center of the formed tubes just after the end of heating inside the forming dies are shown in Figure 25. After contacting the tube with the forming dies, the temperature of the tube rapidly drops and leads to an increase in the flow stress. The cooling ratio is enough even in low internal pressure and the effect of the internal pressure on cooling behavior is slight.

## 5. Conclusions

An integration of hot tube gas forming and die quenching using low pressure sealed-air was developed to form ultra-high strength bulged components. To simplify the control scheme, the required pressure for bulging the tubes was automatically obtained by increasing the temperature of the pressurized air inside the resistance-heated tube without the requirement of a pressure control system. Not only to prevent thinning, but also to adjust the start of bulging, both ends of the tube are axially compressed. Although the axial feed provides materials flow to prevent thinning, the shape, accuracy and thinning ratio were remarkably influenced by the start time of axial feeding. Utilization of sealed-air hot tube gas forming even in low initial internal pressure is significantly effective to produce ultra-high strength hollow products. The Vickers hardness of the die quenched tube by a low pressure of less than 3 MPa was reached to above 95% of the water quenched one.

Although the ultra-high strength steel sheets are useful for reducing the weight of automobiles, the welding process is required. Therefore, the tubular components are attractive as not only do they reduce the weight, but also for packaging purposes as they eliminate the requirement of the overlap portion for spot welding. However, forming and change in the cross-section of the tubes are challengeable, especially for future lightweighting and the production of complex shape hollow components. Although gas forming processes promote the bulging formability of tubes, costly high pressure equipment is required for them. Moreover, compressing the gas with high pressure requires high energy during its preparation. The present gas forming process using low pressure sealed-air has led to the simplification of gas forming equipment and the ability to obtain ultra-high strength hollow products without additional heat treatments, even at quite a low internal pressure, and thus, the industrial applications have become comparatively high. However, it is desirable to develop more useful approaches for producing components having sharp corners or small corner radiuses by a combination of simultaneous bulging and compression processes using low pressure sealed-air. Moreover, the effect of the initial tube shape such as a box-shaped tube or curved tube, and the influence of the temperature drop on die quenching in both axisymmetric and non-axisymmetric gas bulging of tubes, should still be studied. 

## Figures and Tables

**Table 1:** Conditions of sealed-air tube gas forming.

Parameter	Value
Current density (A/mm^2^)	30 (5.6 kA)
Heating time (s)	11.5
Heating temperature (°C)	1050
Distance between electrodes (mm)	100
Initial internal air pressure *p*_0_ (MPa)	0–2.5
Velocity of axial feeding (mm/s)	25
Capacity of axial force (kN)	20
Axial feeding s (mm)	0–15

**Table 2:** Chemical composition of quenchable steel tube (wt%).

C	Si	Mn	P	S	Cr	B	Fe
0.187	0.159	1.37	0.0170	0.0040	0.29	0.0039	Bal.
